# Current status and quality of radiomics studies for predicting outcome in acute ischemic stroke patients: a systematic review and meta-analysis

**DOI:** 10.3389/fneur.2023.1335851

**Published:** 2024-01-02

**Authors:** Jinfen Kong, Danfen Zhang

**Affiliations:** Department of Radiology, Yuhuan Second People's Hospital, Yuhuan, Taizhou, Zhejiang, China

**Keywords:** acute ischemic stroke, radiomics, prognosis prediction, reperfusion prediction, systematic review and meta-analysis

## Abstract

**Background:**

Pre-treatment prediction of reperfusion and long-term prognosis in acute ischemic stroke (AIS) patients is crucial for effective treatment and decision-making. Recent studies have demonstrated that the inclusion of radiomics data can improve the performance of predictive models. This paper reviews published studies focused on radiomics-based prediction of reperfusion and long-term prognosis in AIS patients.

**Methods:**

We systematically searched PubMed, Web of Science, and Cochrane databases up to September 9, 2023, for studies on radiomics-based prediction of AIS patient outcomes. The methodological quality of the included studies was evaluated using the phase classification criteria, the radiomics quality scoring (RQS) tool, and the Prediction model Risk Of Bias Assessment Tool (PROBAST). Two separate meta-analyses were performed of these studies that predict long-term prognosis and reperfusion in AIS patients.

**Results:**

Sixteen studies with sample sizes ranging from 67 to 3,001 were identified. Ten studies were classified as phase II, and the remaining were categorized as phase 0 (*n* = 2), phase I (*n* = 1), and phase III (*n* = 3). The mean RQS score of all studies was 39.41%, ranging from 5.56 to 75%. Most studies (87.5%, 14/16) were at high risk of bias due to their retrospective design. The remaining two studies were categorized as low risk and unclear risk, respectively. The pooled area under the curve (AUC) was 0.88 [95% confidence interval (CI) 0.84–0.92] for predicting the long-term prognosis and 0.80 (95% CI 0.74–0.86) for predicting reperfusion in AIS.

**Conclusion:**

Radiomics has the potential to predict immediate reperfusion and long-term outcomes in AIS patients. Further external validation and evaluation within the clinical workflow can facilitate personalized treatment for AIS patients. This systematic review provides valuable insights for optimizing radiomics prediction systems for both reperfusion and long-term outcomes in AIS patients.

**Systematic review registration:**

https://www.crd.york.ac.uk/prospero/display_record.php?ID=CRD42023461671, identifier CRD42023461671.

## 1 Introduction

Stroke is a major global health issue, characterized by high disability, fatality, and recurrence rates (1). It brings a heavy burden on individuals, families, and society (2). Despite the decrease in incidence and prevalence of stroke globally, China still has a rising number of new cases, with 250 million reported every year (3–5). Acute ischemic stroke (AIS), accounts for 60%-80% of strokes (6), as defined by sudden neurological deficits caused by focal cerebral ischemia (7). Early intervention is critical to improve outcomes in AIS patients. The goal of treatment is to achieve recanalization and reperfusion of the ischaemic penumbra (8, 9). However, many individuals still have severe residual impairments after treatments. Therefore, it is important to accurately predict the prognosis of stroke and determine the long-term treatment plan for patients (8, 10).

Rapid and accurate prediction of outcomes such as success reperfusion and long-term prognosis is vital for effective treatment selection. Traditional predictive models often rely on clinical and conventional image parameters, but their predictive power has limitations. Radiomics is a rapidly evolving field of research that involves extracting quantitative metrics, known as radiomic features, from medical images (11–13). Computed tomography (CT) and magnetic resonance imaging (MRI) are primary diagnostic tools (14, 15), while their radiomic features also hold great potential for prognosis prediction (16, 17). Given the complexity of radiomics data, machine learning (ML) algorithms play a vital role in its application (18–21), particularly in acute stroke imaging (22, 23).

In the landscape of AIS management, conventional imaging remains a cornerstone in diagnosis. The American Heart Association/American Stroke Association (AHA/ASA) guidelines for the early management of patients with AIS and the Chinese guidelines for the diagnosis and treatment of AIS both include non-contrast CT and conventional MRI as an important part of stroke diagnosis (24, 25). Additionally, techniques like CT perfusion and multimodal MRI are invaluable in guiding treatments such as thrombolytic therapy and endovascular thrombolysis (26–29). However, the rapidly developing field of radiomics is providing new pathways for diagnosis and treatment decision-making. For instance, a recent study illustrated that high-dimension radiomics features derived from CT angiography enhances the accuracy of AIS diagnosis and subtype classification, which is crucial for timely intervention (30). Additionally, another study has shown that radiomic models based on apparent diffusion coefficient (ADC) map are highly effective in identifying the ischemic penumbra, which is important in assessing the normal and ischemic penumbra areas and influencing treatment decisions (31). In terms of prognostic prediction, radiomics studies have seen substantial growth in recent years. Since prognosis in AIS is influenced by various factors, these studies often aim to combine radiomics features with clinical information to more precise predictions of patient outcomes. Although currently not used in clinical practice, the combination of radiomics with established imaging techniques will allow us to take a significant step forward in understanding and processing AIS.

While several studies have evaluated the utility of radiomics in AIS prediction, there is a lack of comprehensive reviews that systematically evaluate the methodological quality and predictive accuracy of these studies. This systematic review and meta-analysis aim to address this gap, focusing on reperfusion and long-term prognosis prediction in AIS patients.

## 2 Methods

This systematic review and meta-analysis strictly adhered to the Preferred Reporting Items for Systematic Reviews and Meta-Analyses 2020 (PRISMA 2020) statement. The PRISMA checklist is provided in Supplementary Table 1. And the protocol is prospectively registered in PROSPERO (CRD4202346167).

### 2.1 Literature search strategy

A comprehensive literature search for potentially relevant articles was conducted in PubMed, Cochorane, and Web of Science databases (WOS), with data retrieval up to September 9, 2023. A researcher (Jinfen Kong) designed the keywords and search strategy of this systematic review, and both subject headings and free words were searched. The complete search strategy can be found in the Supplementary Table 2.

### 2.2 Inclusion and exclusion criteria

#### 2.2.1 Inclusion criteria

(1) Patients were diagnosed with AIS using CT or MRI.
(2) Radiomic features were utilized to predict outcome in AIS patients.
(3) Long-term outcome was measured using the modified Rankin Scale (mRS) or discharge National Institute of Health Stroke Scale (NIHSS).
(4) Reperfusion was assessed using the modified Thrombolysis in Cerebral Infarction (mTICI) scale or other relevant methods.
(5) Study participants were aged 18 and older.
(6) Full-text was available and articles were written in English.

#### 2.2.2 Exclusion criteria

(1) Studies conducted in phantom or animal models.
(2) Case reports or small case series (≤10 patients).
(3) Reviews, poster presentations, letters, or meeting abstracts.
(4) Cerebral hemorrhage resulting from secondary causes such as cerebral trauma or subarachnoid hemorrhage.
(5) Predictive models rely solely on clinical factors without incorporating radiomic features.

### 2.3 Data extraction

All of the retrieved studies were managed using Endnote. After the removal of duplicates through automated and manual processes, two researchers (Jinfen Kong and Danfen Zhang) independently assessed the remaining articles. Preliminary screening was conducted based on titles and abstracts before downloading full texts. Eligible studies that met inclusion criteria were selected after a thorough examination of the full texts.

Prior to data extraction, a standardized data extraction sheet was prepared, including data source, sample size, population, study design, image modality, research question, treatment, software, segmentation, clinical and image features, validation approach, endpoints, reference standard, and classifier model types. Endpoints of interest were long-term prognosis and reperfusion. Long-term prognosis was defined as mRS or discharge NIHSS. Reperfusion was defined as mTICI or other methods. For long-term prognosis, mRS thresholds and follow-up time were recorded. For reperfusion, mTICI thresholds defined as successful reperfusion were recorded. Performance metrics area under the curve (AUC) were extracted.

### 2.4 Quality assessment

The methodological quality of the included studies was independently evaluated by two reviewers (Jinfen Kong and Danfen Zhang) and cross-checked for consistency.

#### 2.4.1 Model quality assessment

The phase classification criteria is a model quality assessment tool (32). The parameters for phase categorization included sample size (<100 or >100), study design (retrospective or prospective), type of validation approach (internal or independent), and the development stage (pre- or post-marketing). The phase classification criteria categorized image mining studies into the discovery science and phases 0–IV.

#### 2.4.2 Radiomics quality assessment

Radiomics quality was assessed using the 16-component Radiomics Quality Score (RQS) tool (33). Each study was assigned a number of points per RQS component and summed to give a total score ranging from −8 to +36. A score of −8 to 0 points corresponds to 0% and 36 points correspond to 100%. The mean score of the two evaluations is presented as a percentage.

#### 2.4.3 Risk of bias assessment

The Risk of Bias (ROB) of included studies was evaluated using the Prediction Model Risk of Bias Assessment Tool (PROBAST) (34). This assessment encompassed four major domains: participants, predictors, outcomes, and statistical analysis, ultimately reflecting overall ROB and applicability. These domains include two, three, six and nine questions, respectively. Questions are answered as either yes/probable yes (Y/PY), no/probably no (N/PN), or no information (NI). If a domain is answered with at least a N/PN, it is considered at high ROB. An overall low ROB rating was achieved only when all four domains were rated as low ROB.

### 2.5 Meta-analysis

Two meta-analyses were performed: (1) A meta-analysis to evaluate the use of radiomics for predicting long-term prognosis, and (2) A meta-analysis to assess the use of radiomics in predicting reperfusion. When multiple models were reported in a study, only the one with the highest AUC was extracted. If multiple validation datasets of the optimal model were reported, we also extracted the highest AUC among them. Meta-analysis was performed on the metrics (AUC) for evaluating machine learning models. If AUC lacked 95% confidence interval (CI) and standard error (SE), we estimated SE based on a study by Debray et al. (35). Given the difference in variables and parameters in ML models, a random-effects model was employed to perform the meta-analysis. R package metafor (v4.4-0) was used for meta-analyses. Heterogeneity was assessed using the *I*^2^ statistic, with low, moderate, and high levels of heterogeneity corresponding to *I*^2^ values of 25, 50, and 75%, respectively (36). Publication bias was examined using funnel plots, Egger's bias test, and Begg's test. Two-sided *p* < 0.05 were considered statistically significant.

### 2.6 Sensitivity analysis

The impact of each study on the overall results was analyzed by excluding one study at a time and recalculating the combined effect size. R package metafor (v4.4-0) was used for sensitivity analysis.

## 3 Results

### 3.1 Study selection

**Figure 1:**
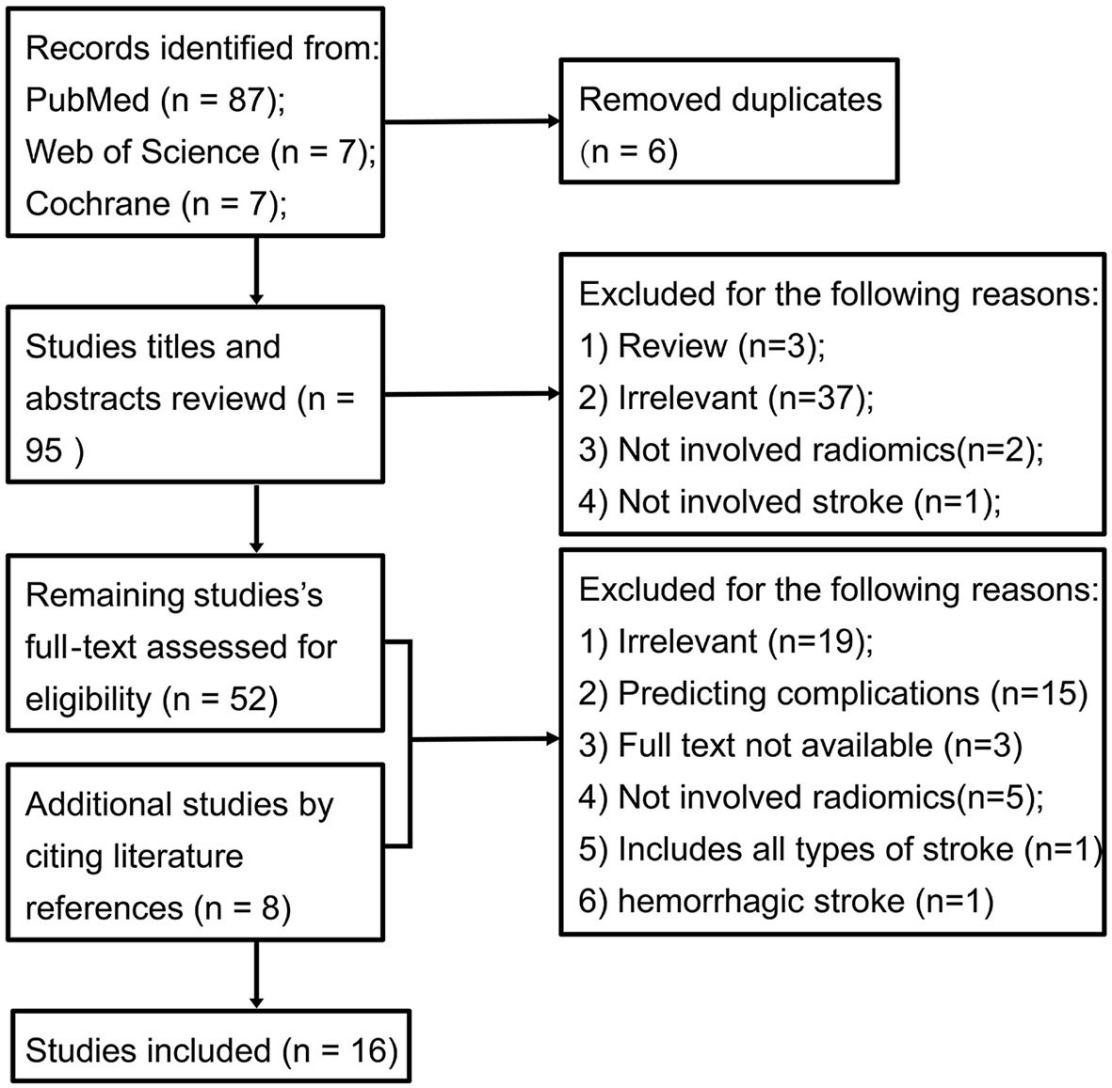
PRISMA flowchart of included studies.

Figure 1 illustrates the PRISMA flowchart outlining the study selection process. Our search strategy initially identified 87 studies from PubMed, 7 from Web of Science (WOS), and 7 from Cochrane (Figure 1 and Supplementary Table 2). After removing 6 duplicates, we screened 95 titles/abstracts. This led to the inclusion of 52 studies and 8 additional cited studies for full-text review. Ultimately, 16 peer-reviewed articles were included in this meta-analysis.

### 3.2 Study characteristics

A total of 16 eligible studies were included in this systematic review (13, 37–51), and their characteristics are presented in Table 1 and Supplementary Table 3. The radiomics modality used in these studies was relatively balanced, with half employing CT image features and the remaining half using MR image features. The majority of studies used baseline images of AIS patients, while two studies (39, 46) included post-treatment images to predict long-term outcome. The treatment strategy in most studies is mechanical thrombectomy (MTB), while the remaining studies employed diverse treatments, including intravenous thrombolytic (IVT) therapy, endovascular treatment (EVT), intravenous recombinant tissue plasminogen activator (IV-rtPA) therapy, and other conventional treatment.

**Table 1:** Included radiomic studies.

**Study ID**	**Participant**	**Training**	**Test**	**Validation**	**External validation**	**Population**	**Study design**	**Imaging modality**
Limin Zhang	240	194		46	No	AIS	Retrospective	Baseline NCCT
Jeremy Hofmeister	156	109		47	Yes	AIS	Retrospective and prospective	Baseline CT, CTA, CTP
Guanmin Quan	190	110		80	Yes	AIS	Retrospective	Baseline MRI (FLAIR, DWI, MRA)
Linna Li	102	81	21		No	AIS	Retrospective	Baseline and post-operative NCCT
Haoyue Zhang	141	122		29	No	AIS	Retrospective	Baseline DWI, FLAIR
Tatsat R. Patel	74	52		22	No	AIS	Retrospective	Baseline CT (CTA, NCCT)
Xing Xiong	256	95		108	Yes	AIS	Retrospective	Baseline CT (NCCT, CTA, CTP)
Lucas A. Ramos	3,001	1,921	600	480	Yes	AIS	Prospective	Baseline CTA
W. Qiu	67	54		13	No	AIS	Retrospective	Baseline NCCT and CTA
Tian-yu Tang	155	84		71	Yes	AIS	Retrospective and prospective	Baseline DWI and PWI
Hui Cui	70	40	30		No	AIS	Retrospective	Baseline MRI
Manon L. Tolhuisen	206	144	41	21	Yes	AIS	Retrospective	Post-treatment DWI at 24 h
Wei Ye	441	309		132	No	AIS	Retrospective	Baseline MRI
Emily W. Avery	677	373		304	Yes	Acute LVO stroke	Retrospective	Baseline CTA
Liang Jiang	1,716	1,256		460	Yes	Acute LVO stroke	Retrospective	Baseline DWI
Huan Yu	148	104	44		No	AIS	Retrospective	Baseline multi-modal MRI

External validation, “Yes” indicates that an external validation dataset was utilized in the study; “No” indicates that no external validation dataset was employed; NM, not mentioned; AIS, acute ischemic stroke; acute LVO stroke, acute anterior large vessel occlusion Stroke; NCCT, non-contrast computed tomography; CTA, CT angiography; CTP, CT perfusion imaging; MRI, magnetic resonance imaging; FLAIR, fluidattenuated inversion recovery; DWI, diffusion Weighted Imaging; MRA, magnetic Resonance Angiography; PWI, perfusion-weighted imaging.

Within these studies, ten are dedicated to predicting long-term prognosis, five focus on reperfusion (37, 40, 41, 43, 51), and one addresses both aspects (42). For long-term prognosis, assessment of the mRS spanned a follow-up period of 3–12 months, while assessments of the NIHSS occurred at discharge or on day 7. Most studies predicting long-term prognosis using mRS employed a threshold of 2 (Table 2). Regarding reperfusion, five out of six studies utilized the mTICI scale as the reference standard, employing thresholds of mTICI ≥ 2b, 2c, or 3 (Table 3).

**Table 2:** Patient follow-up time and stratification cut-off value in long-term prognostic modeling studies.

**Study ID**	**Endpoints**	**Reference standard**	**Follow-up time**	**Cut-off value**
Limin Zhang	Long-term prognosis	mRS	6 months	2
Guanmin Quan	Long-term prognosis	mRS	3 months	2
Linna Li	Long-term prognosis	mRS	3 months	2
Lucas A. Ramos	Reperfusion and long-term prognosis	mRS	3 months	2
Tian-yu Tang	Long-term prognosis	mRS	mRS at day 7 and 3 months	2
Hui Cui	Long-term prognosis	mRS	3 months	(0, 1, 2, 3, 4), multi-categorization task
Manon L. Tolhuisen	Long-term prognosis	mRS	not mentioned	2
Wei Ye	Long-term prognosis	Discharge NIHSS	not involved	(1, 4, 5), multi-categorization task
Emily W. Avery	Long-term prognosis	mRS	3 months	2
Liang Jiang	Long-term prognosis	mRS	3, 6, 12 months	2
Huan Yu	Long-term prognosis	mRS	not mentioned	2

mRS, modified Rankin Scale; NIHSS, National Institute of Health stroke scale.

**Table 3:** Threshold for successful reperfusion.

**Source**	**Endpoints**	**Reference standard**	**Reference standard**
Jeremy Hofmeister	Reperfusion	mTICI	mTICI ≥ 2b
Haoyue Zhang	Reperfusion	mTICI	mTICI ≥ 2c
Tatsat R. Patel	Reperfusion	mTICI	mTICI ≥ 2c
Xing Xiong	Reperfusion	mTICI	mTICI ≥ 2b, or 3
Lucas A. Ramos	Reperfusion and long-term Prognosis	mTICI	mTICI ≥2b
W. Qiu	Reperfusion	mTICI	mTICI ≥2b

mTICI, modified thrombolysis in cerebral infarction.

Among the 16 studies, 12 conducted a combined model that integrated both clinical variables and radiomics features. Notably, 11 of these studies performed a comparation between models using only clinical features and models incorporating radiomics features. While 4 of these studies (41–43, 46) did not observe a significant difference in predictive performance, the remaining 7 (13, 38, 39, 44, 47–49) demonstrated that the inclusion of radiomics significantly improved the predictive ability for AIS prognosis. Moreover, among these 7 studies, 3 (38, 39, 44) directly compared the performance of the radiomics model with the clinical model, and all found that the radiomics model was superior. Additionally, one study (13) included conventional MRI factors like lesion optical densities (ODs), Fazekas scores, and admission DWI-ASPECTS, alongside clinical features. In this case, the AUC of the radiomics model was better than both the pure clinical model and the clinical + conventional MRI model.

The included studies were published between 2018 and 2023, with seven of them all published in 2023, indicating a huge explosion over these years. This trend highlights the increasing importance of radiomics in the field of prognostic prediction for patients with AIS.

### 3.3 Quality analysis

The sample size of the included studies ranged from 67 to 3,001, with thirteen studies (81.3%) enrolling more than 100 patients. Most studies were retrospective (81.3%), while one study (42) was prospective, and two studies (37, 44) employed a combination of retrospective training and prospective validation. Validation analysis was conducted in all studies, with seven studies using external datasets, seven studies employing the cross-validation method, and only three relying solely on internal datasets. According to the phase classification criteria, ten studies (62.5%) were classified as phase II, and the remaining were distributed as phase 0 (*n* = 2), phase I (*n* = 1), and phase III (*n* = 3) (Table 4).

**Table 4:** Summary of the quality of included studies.

**Study ID**	**Phase**	**RQS score (%)**	**ROB**
Limin Zhang	II	44.44	High
Jeremy Hofmeister	III	47.22	High
Guanmin Qua	II	36.11	High
Linna Li	II	33.33	High
Haoyue Zhang	II	36.11	High
Tatsat R. Patel	I	36.11	High
Xing Xiong	II	36.11	High
Lucas A. Ramos	III	75.00	Low
W. Qiu	0	38.89	High
Tian-yu Tang	III	52.78	Unclear
Hui Cui	0	30.56	High
Manon L. Tolhuisen	II	5.56	High
Wei Ye	II	27.78	High
Emily W. Avery	II	47.22	High
Liang Jiang	II	52.78	High
Huan Yu	II	30.56	High

Phase, phase classification criteria; RQS, radiomics quality score; ROB, risk of bias.

**Figure 2:**
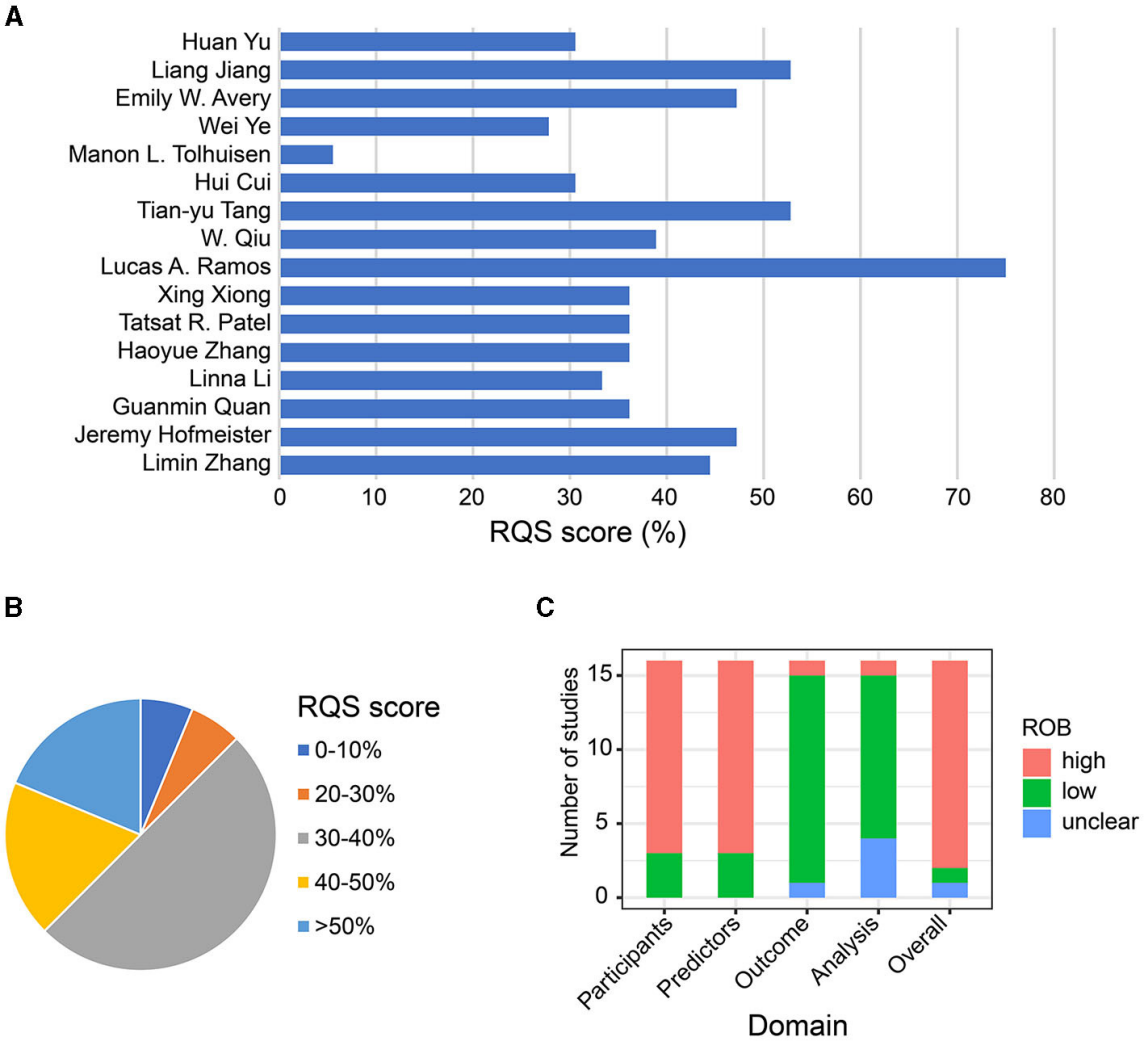
Quality assessment of included studies. **(A)** Bar chart of the mean score of each study according to the radiomics quality scoring tool. **(B)** Pie chart of the mean score of studies according to the radiomics quality scoring tool. **(C)** Evaluation of Risk of bias.

In terms of radiomic-specific quality, Table 4 and Supplementary Table 4 presents the RQS scores of all included studies. And the individual score for each RQS items, for each of our two evaluators are shown in Supplementary Table 5 and Supplementary Figure 1. The mean score across all 16 studies was 39.41% (range 5.56–75%). Most studies scored within 30–40%, with only one study receiving a quality score of <10% (46) (Figures 2A, B). Most studies reported well-documented image acquisition protocols, nine studies (56.3%) mentioned that image segmentation was performed by 2 or more physicians. None addressed inter-scanner differences or vendor-dependent features. All studies acquired images from baseline scans except one study (46) used post-operative images, and another one (39) used both pre- and post-operative images. Feature dimension reduction was performed in most studies, except for one (46). Ten (62.5%) studies incorporated clinical features into radiomic models, and three of these studies suggested that this integration improved predictive performance. Clinical characteristics included in models were age, gender, age, sex, baseline NIHSS, baseline mRS, dyslipidemia, penetrating artery infarction, hypertension, previous stroke, ASPECTS, time since stroke (TSS), time to treatment (TTT), and more. The correlation between clinical factors and radiomic features was discussed in six (37.5%) studies. Only four studies conducted cut-off analysis using nomograms to assess risks. For model assessment, discrimination statistics were typically provided, whereas calibration statistics were less mentioned. Validation of radiomics signatures was performed in all studies, with 7 studies (43.8%) employing external datasets, and 7 using the cross-validation method. However, only one study (42) was prospectively registered. Regarding the clinical utility, 7 studies compared their models with the gold standard, indicating potential clinical utility. But none performed cost-effectiveness analysis. In terms of open science and data, two studies (42, 48) obtained radiomic features on a set of representative regions of interest (ROIs), with the calculated features and code being open access (Figures 2A, B).

For risk of bias (ROB), there are 13 of 16 models originated from retrospective studies, which carried a high risk of bias (ROB). The remaining three studies employed prospective validation, resulting in a low ROB (Table 4 and Supplementary Table 6). In terms of the assessment of predictors domain, 13 studies were rated at high ROB due to the potential bias in retrospective studies where researchers knew both predictors and data results. For the outcome domain, 14 studies had a low ROB, while one model had an unclear ROB (43), and another one had a high ROB (46). Lastly, as for the analysis domain, one study (37) had a high ROB because it selected predictors based solely on univariable analysis, and four studies (38, 40, 44, 46) had unclear ROB due to incomplete disclosure of how missing data were handled, failure to indicate whether data complexity was considered, lack of explanation on how overfitting was avoided, or not illustrating the weight of factors. The summary of ROB evaluation is presented in Figure 2C.

### 3.4 The value of radiomics in predicting AIS outcome

**Figure 3:**
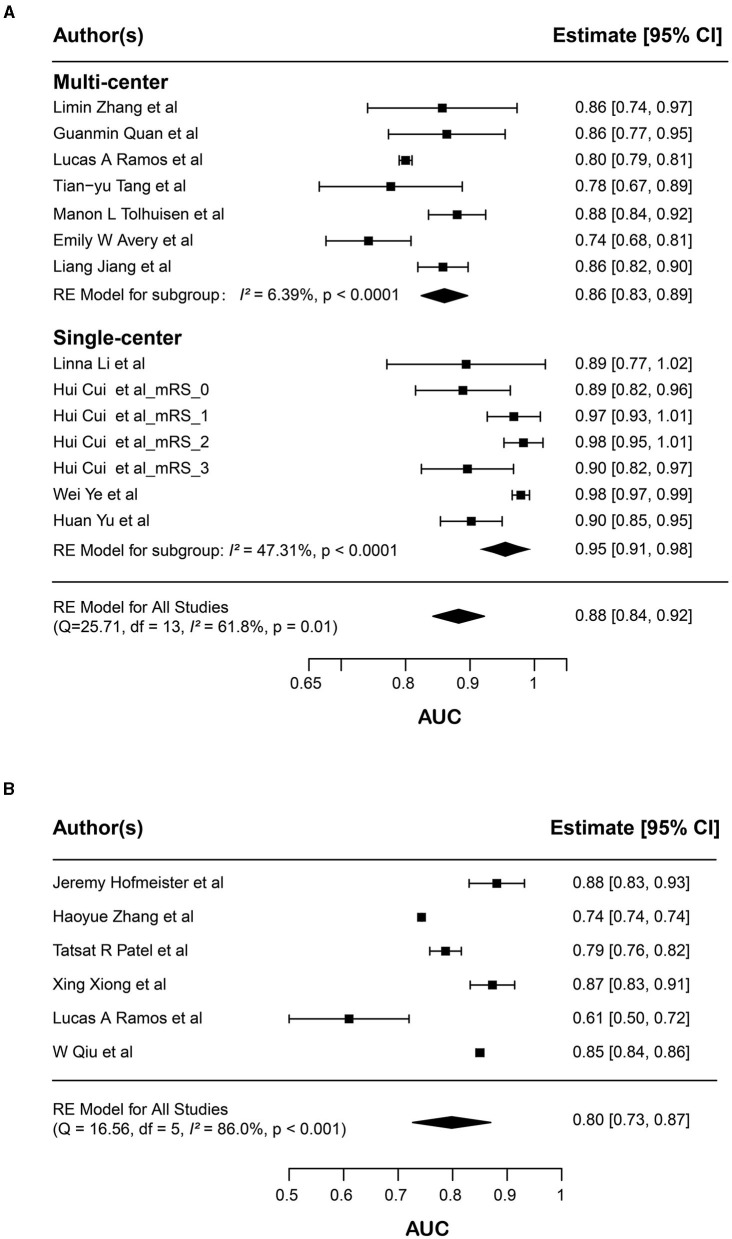
Forest plots of the predictive performance of radiomics models. Forest plots showed the predictive performance of radiomics models in **(A)** long-term prognosis (mRS) and **(B)** immediate reperfusion (mTICI) of AIS patients. For long-term prognosis, the results of subgroup analysis according to single-center or multicenter factors are shown; Area under curve (AUC) for each study is presented as a black dot, with the horizontal line indicating the 95% confidence interval (CI). The pooled result for all studies is presented as a black diamond.

To evaluate the prognostic value of radiomics in AIS patients, we conducted two separate meta-analyses. The first meta-analysis focused on models designed to predict long-term prognosis in AIS patients, involving 14 radiomics models extracted from 11 studies. From these studies, we selected the best-performing model from 10 of them. Additionally, one study (45) implemented a distinctive approach by stratifying the mRS into 0, 1, 2, 3, and 4 and constructing an individualized model for each mRS score, where the model with mRS = 4 had a SE = 0 and was therefore excluded. Consequently, a total of 14 models were incorporated into the first meta-analysis. The result showed a pooled AUC of 0.883, calculated using a random-effects model, with a 95% CI ranging from 0.844 to 0.921 (*p*-value = 0.01) (Figure 3A). The *I*^2^ statistic is 61.8% which indicates high heterogeneity among these studies.

To investigate the potential source of heterogeneity, we performed subgroup analysis, and found that multi-center or single-center is a significant contributor to the overall heterogeneity within the dataset. When analyzing the single-center subgroup, the pooled AUC was 0.95 (95% CI 0.91–0.98, *p* < 0.0001), and the *I*^2^ value was reduced to 47.31%. When analyzing the multi-center subgroup, the pooled AUC was 0.86 (95% CI 0.83–0.89, *p* < 0.0001), and the *I*^2^ value was markedly reduced to 6.39%, indicating a relatively lower heterogeneity (Figure 3A).

In the second meta-analysis, which included six radiomic studies that focused on reperfusion prediction, the pooled AUC was 0.800 (95%CI 0.734–0.863, *p* < 0.001) (Figure 3B). And the *I*^2^ statistic also suggested considerable heterogeneity in the AUC (*I*^2^ = 86.0%). We investigated potential factors that might contribute to the heterogeneity. However, variables such as multi-center or single-center, imaging modality, experimental design, location, and treatment were not able to explain the heterogeneity very well.

In conclusion, these results showed that the synthesized AUCs of the radiomics models are notably high. While some unexplained heterogeneity remains, radiomics exhibits promise in predicting long-term prognosis and reperfusion outcomes in AIS patients.

### 3.5 Publication bias

**Figure 4:**
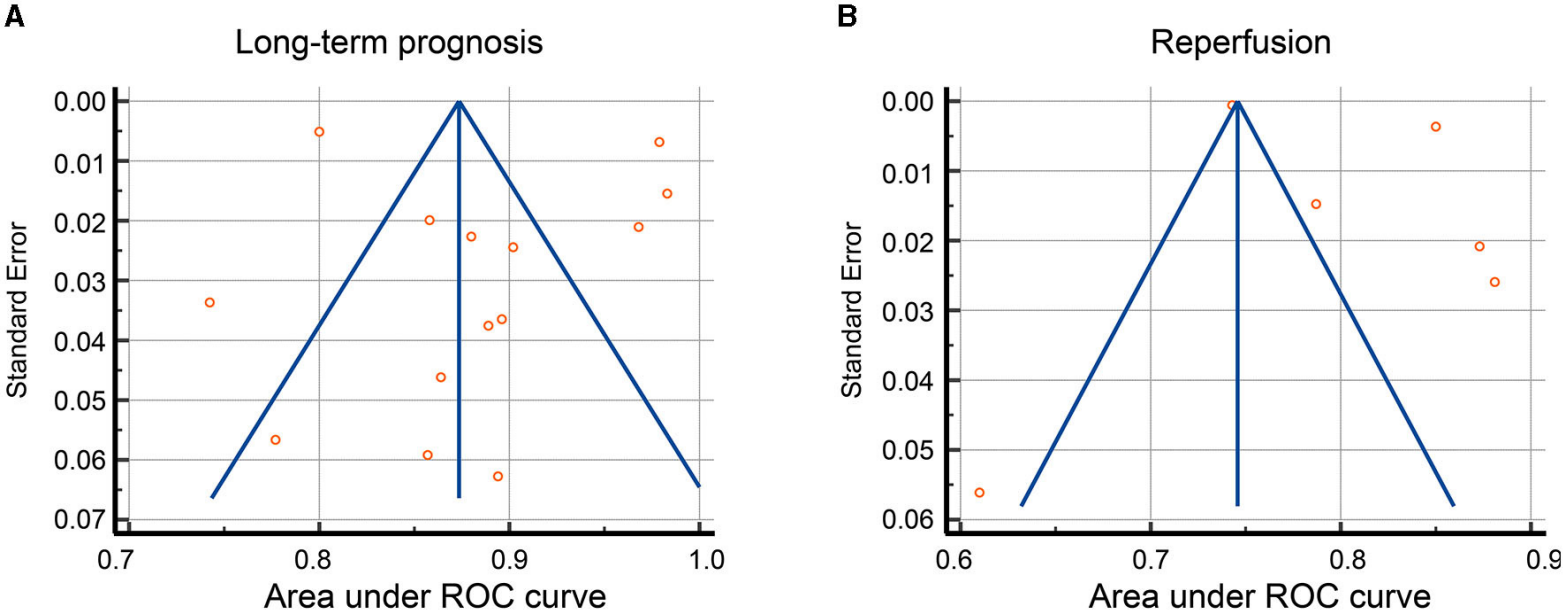
Funnel plot for publication bias of AUC. Funnel plot showed the publication bias of AUC in **(A)** long-term prognosis (mRS) and **(B)** immediate reperfusion (mTICI) of AIS patients.

In the meta-analysis of AUC, no publication bias was detected for predicting both long-term outcome and reperfusion after stroke (Figures 4A, B). Begg's test results further confirmed this observation, with *p*-value = 0.25 for the prediction of long-term outcomes and *p*-value = 0.85 for the prediction of reperfusion.

### 3.6 Robustness of meta-analysis

To demonstrate the robustness of meta-analysis, we performed sensitivity analyses by excluding individual study at a time and recalculating the combined AUC. For long-term prognosis prediction, the synthesized AUC values ranged from 0.832 to 0.949, with standard errors ranged from 0.0036 to 0.0052, which is closely matching the overall values (Table 5). For reperfusion prediction, the synthesized AUC values ranged from 0.743 to 0.847, and standard errors ranged from 0.0006 to 0.0035, which was also close to the overall values (Table 6). These results suggest that these two meta-analysis results are highly robust and are not significantly influenced by any individual study.

**Table 5:** Sensitivity analyses for long-term prognosis prediction.

**Excluded study**	**Leave-one-out AUC**	**Leave-one-out SE**
Limin Zhang	0.8735	0.0036
Guanmin Quan	0.8735	0.0036
Lucas A. Ramos	0.9485	0.0052
Tian-yu Tang	0.8739	0.0036
Manon L. Tolhuisen	0.8733	0.0037
Emily W. Avery	0.875	0.0036
Liang Jiang	0.874	0.0037
Linna Li	0.8734	0.0036
Hui Cui (mRS = 0)	0.8733	0.0036
Hui Cui (mRS = 1)	0.8706	0.0037
Hui Cui (mRS = 2)	0.8671	0.0037
Hui Cui (mRS = 3)	0.8733	0.0036
Wei Ye	0.8323	0.0043
Huan Yu	0.8728	0.0037

AUC, area under curve; SE, standard error.

**Table 6:** Sensitivity analyses for reperfusion prediction.

**Excluded study**	**Leave-one-out AUC**	**Leave-one-out SE**
Jeremy Hofmeister	0.746	0.0006
Haoyue Zhang	0.847	0.0035
Tatsat R. Patel	0.746	0.0006
Xing Xiong	0.746	0.0006
Lucas A. Ramos	0.746	0.0006

AUC, area under curve; SE, standard error.

## 4 Discussion

The increasing popularity of radiomics prediction for assessing AIS patient outcomes is evident from the growing number of studies in recent years. This systematic review and meta-analysis identified 16 studies employing radiomic techniques to predict reperfusion and prognosis in AIS patients. Remarkably, radiomics model demonstrated promising performance, with pooled AUC values of 0.883 for long-term prognosis and 0.800 for reperfusion prediction, validating its potential value in AIS management.

Regarding the quality of studies in our meta-analysis, the average RQS was 14, representing 39.41% of the total RQS, with a median score of 13, corresponding to 36.11% of the total RQS. Most studies scored between 30 to 40%, while two studies scored below 30%, with one as low as 5.56%. Upon comparison with radiomics meta-analyses in other fields, we observed that the quality of radiomics studies is a widely concern. For instance, a review of cholangiocarcinoma encompassing 38 original studies reported a median RQS of 9, amounting to just 25.0% of the total RQS (52). Similarly, a review focusing on ovarian imaging, which included 63 studies, found a median RQS of 6, corresponding to 30.6% of the total RQS, indicating lower scoring (53). Another meta-analysis covering 57 ovarian cancer studies reported an average RQS of 30.7%, which is also considered unsatisfactory (54). Reviews in other areas of radiomics, such as prostate cancer (55), meningiomas (56), nasopharyngeal carcinoma (57), and cardiovascular fields (58), have also highlighted concerns regarding the quality of machine learning and radiomics research, underscoring the general insufficiency in the scientific rigor of radiomics studies (59). This draws attention to the need for a more standardized approach in the radiomics workflow before its integration into clinical practice, to enhance the quality and reliability of findings, thereby ensuring their utility and applicability in clinical settings.

Incorporating clinical characteristics into radiomics models in most cases highlights the importance of a comprehensive approach. Integrating a patient's clinical and radiomic profile can improve predictive accuracy. In fact, several studies demonstrated enhanced performance of prediction after integration, highlighting the advantages of using multidimensional information.

These findings emphasize the increasing acceptance and potential of radiomics in enhancing clinical decision-making for AIS patients. When these high-dimensional data are combined with machine learning models, they have the potential to optimize personalized treatment approaches. Such objective and time-sensitive risk stratification strategy can help with treatment decisions and enable tele-stroke assessment of patients. Particularly in the absence of reliable clinical information at the time of admission, models solely using radiomics features can serve as a valuable prognostication tool.

### 4.1 Limitation

A principal limitation concerns the quality assessment of included studies. The RQS tool is widely used for evaluating the quality of radiomics research. However, a recent study has highlighted the challenges in correctly understanding and implementing RQS to allocate reproducible scores (60), while inappropriate methodological quality assessment can lead to bias in quality assessments. Indeed, our study encountered this inter-rater reliability issue as well, particularly in the items of calibration statistics, validation, and open science and data, where our two evaluators assigned inconsistent scores for 6 included studies (Supplementary Table 5 and Supplementary Figure 1). We analyzed that these inconsistencies arose from the different expertise of the two raters in medical statistics and machine learning, affecting calibration statistics and open science and data, and varied academic experiences in radiology impacting validation item. Our current approach to address these inconsistencies is to average the divergent scores. However, we recognize that this simplistic method is a compromise and reflects a broader challenge faced by researchers using RQS tool. A robust and reproducible scoring tool for radiomics research is crucial for our study and for the field of radiomics. We eagerly anticipate the development of more standardized tools for evaluating radiomics research, which would not only aid our meta-analysis but also guide future studies in radiomics field.

Another critical limitation is evident in the current landscape of AIS radiomics studies. Firstly, for when predicting reperfusion, we observe significant heterogeneity among studies, which could not solely explained by single-center vs. multicenter design. Variations in study populations, methodologies, and the choice of utilizing first-pass effect recirculation results or multiple recirculation results for predicting MTB also could contribute to the inter-study heterogeneity. Indeed, previous studies have emphasized that radiomics studies are heterogeneous in various areas (52–55, 59, 61, 62). Subgroup analysis may partially address this heterogeneity. However, a comprehensive assessment of these factors will require more published studies. Moreover, due to the different parameters used in each article, we extracted AUC for the meta-analysis, as it was reported in all 16 studies. However, for a more comprehensive analysis, parameters such as model accuracy and precision need to be evaluated. Regrettably, due to constraints imposed by the original studies, we were unable to conduct a comprehensive evaluation using these additional parameters.

Additionally, in studies featuring multiple radiomics models, we selected the best-performing model for each. This approach, while enhancing comparability across studies, introducing a selection bias, potentially leading to overly optimistic estimates. We chose the best-performing models based on their ability to provide consistent and comparable benchmarks across studies. However, this necessitates a cautious interpretation of our findings, recognizing that it may limit the overall generalizability of the results. Future research would benefit from incorporating a broader range of models to provide a deeper understanding of predictive capabilities in the field.

Moreover, the dominance of retrospective studies raises concerns about selection bias and potentially overestimate of model performance. The lack of consideration for inter-scanner differences or vendor-dependent features further impact image quality and feature extraction. To enhance consistency and reproducibility, it is crucial to establish a unified protocol for image acquisition and processing. Moving forward, future studies should prioritize prospective, multicentric collaborations to validate and generalize the predictive power of radiomics models across diverse populations and clinical settings.

## 5 Conclusions

This review provides a quality assessment and meta-analysis of studies that using radiomics features to predict outcome in AIS patients, highlighting the potential of radiomics-based predictive models. As the field continues to grow, integrating radiomics-based machine learning models into clinical pathways is expected to improve personalized care of AIS patients. Future efforts should further leverage this potential to optimize patient outcomes.

## Data availability statement

The original contributions presented in the study are included in the article/Supplementary material, further inquiries can be directed to the corresponding author.

## Author contributions

JK: Conceptualization, Investigation, Methodology, Project administration, Software, Supervision, Validation, Writing – original draft, Writing – review & editing. DZ: Data curation, Formal analysis, Software, Validation, Visualization, Writing – review & editing.
