# Supplementary material for: Current status and quality of radiomics studies for predicting outcome in acute ischemic stroke patients: a systematic review and meta-analysis
Source: Front Neurol. 2024 Jan 2;14:1335851. doi: 10.3389/fneur.2023.1335851 (PMC10789857; doi:10.3389/fneur.2023.1335851)
Supplement: Supplementary file 1 [file Data_Sheet_1.docx]

Supplementary Material

Current status and quality of radiomics studies for predicting outcome in acute ischemic stroke patients: a systematic review and meta-analysis

Jinfen Kong^1^*, Danfen Zhang^1^

*** Correspondence:** Jinfen Kong: [kongjinfen429@163.com](mailto:kongjinfen429@163.com)

# Supplemental table 1. The PRISMA 2020 checklist

| **Section and Topic** | **Item #** | **Checklist item** | **Location where item is reported** |
| --- | --- | --- | --- |
| **TITLE** | | |  |
| Title | 1 | Identify the report as a systematic review. | Page 1 |
| **ABSTRACT** | | |  |
| Abstract | 2 | See the PRISMA 2020 for Abstracts checklist. | Page 1 |
| **INTRODUCTION** | | |  |
| Rationale | 3 | Describe the rationale for the review in the context of existing knowledge. | Page 2 |
| Objectives | 4 | Provide an explicit statement of the objective(s) or question(s) the review addresses. | Page 2 |
| **METHODS** | | |  |
| Eligibility criteria | 5 | Specify the inclusion and exclusion criteria for the review and how studies were grouped for the syntheses. | Page 3 |
| Information sources | 6 | Specify all databases, registers, websites, organisations, reference lists and other sources searched or consulted to identify studies. Specify the date when each source was last searched or consulted. | Page 2 |
| Search strategy | 7 | Present the full search strategies for all databases, registers and websites, including any filters and limits used. | Supplementary Table 2 |
| Selection process | 8 | Specify the methods used to decide whether a study met the inclusion criteria of the review, including how many reviewers screened each record and each report retrieved, whether they worked independently, and if applicable, details of automation tools used in the process. | Page 2 |
| Data collection process | 9 | Specify the methods used to collect data from reports, including how many reviewers collected data from each report, whether they worked independently, any processes for obtaining or confirming data from study investigators, and if applicable, details of automation tools used in the process. | Pages 3-4 |
| Data items | 10a | List and define all outcomes for which data were sought. Specify whether all results that were compatible with each outcome domain in each study were sought (e.g. for all measures, time points, analyses), and if not, the methods used to decide which results to collect. | Pages 3 |
|  | 10b | List and define all other variables for which data were sought (e.g. participant and intervention characteristics, funding sources). Describe any assumptions made about any missing or unclear information. | Pages 4 |
| Study risk of bias assessment | 11 | Specify the methods used to assess risk of bias in the included studies, including details of the tool(s) used, how many reviewers assessed each study and whether they worked independently, and if applicable, details of automation tools used in the process. | Pages 4 |
| Effect measures | 12 | Specify for each outcome the effect measure(s) (e.g. risk ratio, mean difference) used in the synthesis or presentation of results. | Pages 4 |
| Synthesis methods | 13a | Describe the processes used to decide which studies were eligible for each synthesis (e.g. tabulating the study intervention characteristics and comparing against the planned groups for each synthesis (item #5)). | Page 4 |
|  | 13b | Describe any methods required to prepare the data for presentation or synthesis, such as handling of missing summary statistics, or data conversions. | NA |
|  | 13c | Describe any methods used to tabulate or visually display results of individual studies and syntheses. | Page 4 |
|  | 13d | Describe any methods used to synthesize results and provide a rationale for the choice(s). If meta-analysis was performed, describe the model(s), method(s) to identify the presence and extent of statistical heterogeneity, and software package(s) used. | Page 4 |
|  | 13e | Describe any methods used to explore possible causes of heterogeneity among study results (e.g. subgroup analysis, meta-regression). | Page 4 |
|  | 13f | Describe any sensitivity analyses conducted to assess robustness of the synthesized results. | Page 4 |
| Reporting bias assessment | 14 | Describe any methods used to assess risk of bias due to missing results in a synthesis (arising from reporting biases). | Page 4 |
| Certainty assessment | 15 | Describe any methods used to assess certainty (or confidence) in the body of evidence for an outcome. | Page 4 |
| **RESULTS** | | |  |
| Study selection | 16a | Describe the results of the search and selection process, from the number of records identified in the search to the number of studies included in the review, ideally using a flow diagram. | Page 5 and Supplementary Table 2 |
|  | 16b | Cite studies that might appear to meet the inclusion criteria, but which were excluded, and explain why they were excluded. | Page 5 |
| Study characteristics | 17 | Cite each included study and present its characteristics. | Page 5 |
| Risk of bias in studies | 18 | Present assessments of risk of bias for each included study. | Pages 5-6 |
| Results of individual studies | 19 | For all outcomes, present, for each study: (a) summary statistics for each group (where appropriate) and (b) an effect estimate and its precision (e.g. confidence/credible interval), ideally using structured tables or plots. | Page 6 and Figure 3 |
| Results of syntheses | 20a | For each synthesis, briefly summarise the characteristics and risk of bias among contributing studies. | Pages 6-7 |
|  | 20b | Present results of all statistical syntheses conducted. If meta-analysis was done, present for each the summary estimate and its precision (e.g. confidence/credible interval) and measures of statistical heterogeneity. If comparing groups, describe the direction of the effect. | Pages 6-7 |
|  | 20c | Present results of all investigations of possible causes of heterogeneity among study results. | Pages 6-7 |
|  | 20d | Present results of all sensitivity analyses conducted to assess the robustness of the synthesized results. | Page 7 |
| Reporting biases | 21 | Present assessments of risk of bias due to missing results (arising from reporting biases) for each synthesis assessed. | Page 6 |
| Certainty of evidence | 22 | Present assessments of certainty (or confidence) in the body of evidence for each outcome assessed. | Pages 6-7 |
| **DISCUSSION** | | |  |
| Discussion | 23a | Provide a general interpretation of the results in the context of other evidence. | Pages 7-8 |
|  | 23b | Discuss any limitations of the evidence included in the review. | Page 8 |
|  | 23c | Discuss any limitations of the review processes used. | Page 8 |
|  | 23d | Discuss implications of the results for practice, policy, and future research. | Page 8 |
| **OTHER INFORMATION** | | |  |
| Registration and protocol | 24a | Provide registration information for the review, including register name and registration number, or state that the review was not registered. | Page 2 |
|  | 24b | Indicate where the review protocol can be accessed, or state that a protocol was not prepared. | Page 2 |
|  | 24c | Describe and explain any amendments to information provided at registration or in the protocol. | NA |
| Support | 25 | Describe sources of financial or non-financial support for the review, and the role of the funders or sponsors in the review. | Page 9 |
| Competing interests | 26 | Declare any competing interests of review authors. | Page 8 |
| Availability of data, code and other materials | 27 | Report which of the following are publicly available and where they can be found: template data collection forms; data extracted from included studies; data used for all analyses; analytic code; any other materials used in the review. | Page 9 |

# Supplemental table 2. Literature search strategy

**1.Pubmed**

| **ID** | **Query** | **Results** |
| --- | --- | --- |
| #1 | Stroke[MeSH] | 173980 |
| #2 | (((((((Stroke[Title/Abstract]) OR (Strokes[Title/Abstract])) OR (Cerebrovascular Accident[Title/Abstract])) OR (Cerebrovascular Accidents[Title/Abstract])) OR (Ischemic Stroke[Title/Abstract])) OR (Ischemic Strokes[Title/Abstract])) OR (Cerebral Stroke[Title/Abstract])) OR (Cerebral Strokes[Title/Abstract]) | 327413 |
| #3 | #1 OR #2 | 366029 |
| #4 | (Radiomic[Title/Abstract]) OR (Radiomics[Title/Abstract]) | 9097 |
| #5 | (((((((magnetic resonance imaging[Title/Abstract]) OR (magnetic resonance[Title/Abstract])) OR (MRI[Title/Abstract])) OR (MR[Title/Abstract])) OR (computed tomography[Title/Abstract])) OR (CT[Title/Abstract])) OR (positron emission tomography[Title/Abstract])) OR (PET[Title/Abstract]) | 1,295,995 |
| #6 | #4 AND #5 | 7136 |
| #7 | #3 AND #6 | 87 |

**2.Web of science**

| **ID** | **Search** | **Hits** |
| --- | --- | --- |
| #1 | Stroke (Topic) or Strokes (Topic) or Cerebrovascular Accident (Topic) or Cerebrovascular Accidents (Topic) or Ischemic Stroke (Topic) or Ischemic Strokes (Topic) or Cerebral Stroke (Topic) or Cerebral Strokes (Topic) and Preprint Citation Index (Exclude – Database) | 627536 |
| #2 | Radiomic or Radiomics (Topic) and magnetic resonance imaging (Topic) or magnetic resonance (Topic) or MRI (Topic) or MR (Topic) or computed tomography (Topic) or CT (Topic) or positron emission tomography (Topic) or PET (Topic) and Preprint Citation Index (Exclude – Database) (Topic) | 659 |
| #3 | #1 AND #2 | 7 |

**3.Cochrane**

| **ID** | **Search** | **Hits** |
| --- | --- | --- |
| #1 | MeSH descriptor: [Stroke] explode all trees | 15092 |
| #2 | (Stroke):ti,ab,kw OR (Strokes):ti,ab,kw OR (Cerebrovascular Accident):ti,ab,kw OR (Cerebrovascular Accidents):ti,ab,kw (Word variations have been searched) | 72736 |
| #3 | (Ischemic Stroke):ti,ab,kw OR (Ischemic Strokes):ti,ab,kw OR (Cerebral Stroke):ti,ab,kw OR (Cerebral Strokes):ti,ab,kw (Word variations have been searched) | 22926 |
| #4 | #1 OR #2 OR #3 | 73337 |
| #5 | (Radiomic):ti,ab,kw OR (Radiomics):ti,ab,kw | 613 |
| #6 | (magnetic resonance imaging):ti,ab,kw OR (magnetic resonance):ti,ab,kw OR (MRI):ti,ab,kw OR (MR):ti,ab,kw | 52363 |
| #7 | (computed tomography):ti,ab,kw OR (CT):ti,ab,kw OR (positron emission tomography):ti,ab,kw OR (PET):ti,ab,kw | 100335 |
| #8 | (#5 AND #6) OR (#5 AND #7) | 536 |
| #9 | #4 AND #8 | 7 |

# Supplemental table 3. The PRISMA literature list.

| **Study ID** | **Treatment** | **Software** | **Segmen-tation** | **Imaging features** | **Validation** | **Endpoints** | **Reference standard** | **Classifier** |
| --- | --- | --- | --- | --- | --- | --- | --- | --- |
| Limin Zhang | conventional treatment | 3D Slicer, Python scikit-learn, SPSS | Manual | Texture features | Cross-validation | long-term prognosis | mRS | SVM |
| Jeremy Hofmeister | MTB | 3D Slicer, Python | Manual | First-order statistics, shape, and textural features | Split sample, external | reperfusion | mTICI | SVM |
| Guanmin Quan | IVT, anti-thrombotic therapy, anticoagulant, oral statins, and antiplatelet | ITK-SNAP, Python, SPSS | Manual | Histogram and texture features | Split sample, external | long-term prognosis | mRS | multivariable LR |
| Linna Li | MTB | ITK-SNAP, Python, SPSS, RIAS | Manual | Texture feature | Cross-validation | long-term prognosis | mRS | LR |
| Haoyue Zhang | MTB | Python | Automatic | Histogram and texture features | Cross-validation | reperfusion | mTICI | SVM, KNN, LR, and RF |
| Tatsat R. Patel | MTB | 3D Slicer, Python, ITK-SNAP | Manual | Texture feature | Cross-validation | reperfusion | mTICI | LR |
| Xing Xiong | MTB | ITK-SNAP, Python, SPSS | Manual | Texture feature | Split sample, external | reperfusion | mTICI | NB, RF, LR, linear SVM, radial SVM, and ANN |
| Lucas A. Ramos | EVT | Python | Automatic | Histogram and texture features | Cross-validation | reperfusion and long-term prognosis | mRS and mTICI | RFC, SVM, ANN, XGB, and LR |
| W. Qiu | IVT | 3D Slicer, Matlab, ITK-SNAP | Manual | First-order statistics, Shape and size, texture features | Cross-validation | reperfusion | not mentioned | SVM |
| Tian-yu Tang | IV-rtPA or conventional medical treatments | FMRIB Software Library, Matlab, SPSS, Python, R | Automatic | First-order statistics, Shape and size, texture features | Split sample, external | long-term prognosis | NIHSS and mRS | LR |
| Hui Cui | not mentioned | not mentioned | Manual | Histogram and texture features | Split sample | long-term prognosis | mRS | C -support vector classification model |
| Manon L. Tolhuisen | EVT, best medical management alone, IVT | SPM8 toolbox, ITK-SNAP, Python | Automatic | First-order statistics, shape, and textural features | Split sample, external | long-term prognosis | mRS | SVM |
| Wei Ye | not mentioned | Python, SPSS, ITK-SNAP | Manual | First-order statistics, shape, and textural features | Split sample | long-term prognosis | discharge NIHSS | optimized ensemble of deep learning |
| Emily W. Avery | MTB | FSL software FLIRT tool, Python | Automatic | First-order statistics, and textural features | split sample, cross-validation, and external validation | long-term prognosis | mRS | RF, XGBoost, LR, NB, SVM |
| Liang Jiang | standard stroke treatment | Python, R, ITK-SNAP | Manual | First-order statistics, shape, and textural features | Split sample, external | long-term prognosis | mRS | Cox proportional risk regression |
| Huan Yu | not mentioned | MRIcron, SPSS | Manual | First-order statistics, shape, and textural features | Split sample | long-term prognosis | mRS | SVM, RF, LightGBM, CatBoost, and XGBoost |

MTB, mechanical thrombectomy, IVT, intravenous thrombolytic therapy, EVT, endovascular treatment, IV-rtPA, intravenous recombinant tissue plasminogen activator treatments, NIHSS, National Institute of Health stroke scale, mRS, modified rankin scale, mTICI, modified thrombolysis in cerebral infarction, SVM, support vector machine, LR, logistic regression, KNN, K-Nearest Neighbor, RF, random forest, ANN, artificial neural network, NB, naïve Bayes.

# Supplemental table 4. Summary of the radiomics quality score of included studies

| **Component** | **S1** | **S2** | **S3** | **S4** | **S5** | **S6** | **S7** | **S8** | **S9** | **S10** | **S11** | **S12** | **S13** | **S14** | **S15** | **S16** |
| --- | --- | --- | --- | --- | --- | --- | --- | --- | --- | --- | --- | --- | --- | --- | --- | --- |
| Image protocol quality | 1 | 1 | 1 | 1 | 2 | 1 | 1 | 1 | 1 | 1 | 0 | 0 | 1 | 1 | 2 | 1 |
| Multiple segmentations | 1 | 0 | 1 | 1 | 0 | 0 | 1 | 0 | 1 | 0 | 0 | 1 | 1 | 0 | 1 | 1 |
| Phantom study on all scanners | 0 | 0 | 0 | 0 | 0 | 0 | 0 | 0 | 0 | 0 | 0 | 0 | 0 | 0 | 0 | 0 |
| Imaging at multiple time points | 0 | 0 | 0 | 0 | 0 | 0 | 0 | 0 | 0 | 1 | 0 | 0 | 0 | 0 | 0 | 0 |
| Feature reduction or adjustment for multiple testing | 3 | 3 | 3 | 3 | 3 | 3 | 3 | 3 | 3 | 3 | 3 | -3 | 3 | 3 | 3 | 3 |
| Multivariable analysis with non radiomics features | 1 | 0 | 1 | 1 | 0 | 0 | 1 | 1 | 1 | 0 | 1 | 0 | 1 | 1 | 1 | 0 |
| Detect and discuss biological correlates | 1 | 1 | 0 | 0 | 0 | 1 | 0 | 1 | 0 | 0 | 1 | 1 | 0 | 0 | 0 | 0 |
| Cut-off analyses | 1 | 1 | 0 | 0 | 0 | 0 | 0 | 0 | 0 | 1 | 0 | 0 | 0 | 0 | 1 | 0 |
| Discrimination statistics | 2 | 1 | 1 | 1 | 2 | 2 | 1 | 2 | 2 | 2 | 0 | 0 | 1 | 2 | 2 | 1 |
| Calibration statistics | 2 | 2 | 1 | 1 | 2 | 2 | 1 | 2 | 0 | 2 | 0 | 0 | 1 | 2 | 2 | 1 |
| Prospective study registered in a trial database | 0 | 0 | 0 | 0 | 0 | 0 | 0 | 7 | 0 | 0 | 0 | 0 | 0 | 0 | 0 | 0 |
| Validation | 2 | 2 | 3 | 2 | 2 | 2 | 3 | 5 | 2 | 5 | 2 | 3 | 2 | 3 | 3 | 2 |
| Comparison to ‘gold standard’ | 0 | 2 | 0 | 0 | 0 | 0 | 0 | 2 | 2 | 2 | 2 | 0 | 0 | 2 | 2 | 0 |
| Potential clinical utility | 2 | 2 | 2 | 2 | 2 | 2 | 2 | 2 | 2 | 2 | 2 | 0 | 0 | 2 | 2 | 2 |
| Cost-effectiveness analysis | 0 | 0 | 0 | 0 | 0 | 0 | 0 | 0 | 0 | 0 | 0 | 0 | 0 | 0 | 0 | 0 |
| Open science and data | 0 | 0 | 0 | 0 | 0 | 0 | 0 | 1 | 0 | 0 | 0 | 0 | 0 | 1 | 0 | 0 |
| total points=36=100% | 16 | 17 | 13 | 12 | 13 | 13 | 13 | 27 | 14 | 19 | 11 | 2 | 10 | 17 | 19 | 11 |
| Overall Score (%) | 44.44 | 47.22 | 36.11 | 33.33 | 36.11 | 36.11 | 36.11 | 75.00 | 38.89 | 52.78 | 30.56 | 5.56 | 27.78 | 47.22 | 52.78 | 30.56 |

Study ID: S1, Limin Zhang, S2, Jeremy Hofmeister, S3, Guanmin Quan, S4, Linna Li, S5, Haoyue Zhang, S6, Tatsat R. Patel, S7, Xing Xiong, S8, Lucas A. Ramos, S9, W. Qiu, S10, Tian-yu Tang, S11, Hui Cui, S12, Manon L. Tolhuisen, S13, Wei Ye, S14, Emily W. Avery, S15, Liang Jiang, S16, Huan Yu.

# Supplemental table 5. The individual radiomics quality score by each rater

|  | **S1** | | **S2** | | **S3** | | **S4** | | **S5** | | **S6** | | **S7** | | **S8** | | **S9** | | **S10** | | **S11** | | **S12** | | **S13** | | **S14** | | **S15** | | **S16** | |
| --- | --- | --- | --- | --- | --- | --- | --- | --- | --- | --- | --- | --- | --- | --- | --- | --- | --- | --- | --- | --- | --- | --- | --- | --- | --- | --- | --- | --- | --- | --- | --- | --- |
|  | **R1** | **R2** | **R1** | **R2** | **R1** | **R2** | **R1** | **R2** | **R1** | **R2** | **R1** | **R2** | **R1** | **R2** | **R1** | **R2** | **R1** | **R2** | **R1** | **R2** | **R1** | **R2** | **R1** | **R2** | **R1** | **R2** | **R1** | **R2** | **R1** | **R2** | **R1** | **R2** |
| Image protocol quality | 1 | 1 | 1 | 1 | 1 | 1 | 1 | 1 | 2 | 2 | 1 | 1 | 1 | 1 | 1 | 1 | 1 | 1 | 1 | 1 | 0 | 0 | 0 | 0 | 1 | 1 | 1 | 1 | 2 | 2 | 1 | 1 |
| Multiple segmentations | 1 | 1 | 0 | 0 | 1 | 1 | 1 | 1 | 0 | 0 | 0 | 0 | 1 | 1 | 0 | 0 | 1 | 1 | 0 | 0 | 0 | 0 | 1 | 1 | 1 | 1 | 0 | 0 | 1 | 1 | 1 | 1 |
| Phantom study on all scanners | 0 | 0 | 0 | 0 | 0 | 0 | 0 | 0 | 0 | 0 | 0 | 0 | 0 | 0 | 0 | 0 | 0 | 0 | 0 | 0 | 0 | 0 | 0 | 0 | 0 | 0 | 0 | 0 | 0 | 0 | 0 | 0 |
| Imaging at multiple time points | 0 | 0 | 0 | 0 | 0 | 0 | 0 | 0 | 0 | 0 | 0 | 0 | 0 | 0 | 0 | 0 | 0 | 0 | 1 | 1 | 0 | 0 | 0 | 0 | 0 | 0 | 0 | 0 | 0 | 0 | 0 | 0 |
| Feature reduction or adjustment for multiple testing | 3 | 3 | 3 | 3 | 3 | 3 | 3 | 3 | 3 | 3 | 3 | 3 | 3 | 3 | 3 | 3 | 3 | 3 | 3 | 3 | 3 | 3 | -3 | -3 | 3 | 3 | 3 | 3 | 3 | 3 | 3 | 3 |
| Multivariable analysis with non radiomics features | 1 | 1 | 0 | 0 | 1 | 1 | 1 | 1 | 0 | 0 | 0 | 0 | 1 | 1 | 1 | 1 | 1 | 1 | 0 | 0 | 1 | 1 | 0 | 0 | 1 | 1 | 1 | 1 | 1 | 1 | 0 | 0 |
| Detect and discuss biological correlates | 1 | 1 | 1 | 1 | 0 | 0 | 0 | 0 | 0 | 0 | 1 | 1 | 0 | 0 | 1 | 1 | 0 | 0 | 0 | 0 | 1 | 1 | 1 | 1 | 0 | 0 | 0 | 0 | 0 | 0 | 0 | 0 |
| Cut-off analyses | 1 | 1 | 1 | 1 | 0 | 0 | 0 | 0 | 0 | 0 | 0 | 0 | 0 | 0 | 0 | 0 | 0 | 0 | 1 | 1 | 0 | 0 | 0 | 0 | 0 | 0 | 0 | 0 | 1 | 1 | 0 | 0 |
| Discrimination statistics | 2 | 2 | 1 | 1 | 0 | 2 | 1 | 1 | 2 | 2 | 2 | 2 | 1 | 1 | 2 | 2 | 2 | 2 | 2 | 2 | 0 | 0 | 0 | 0 | 1 | 1 | 2 | 2 | 2 | 2 | 1 | 1 |
| Calibration statistics | 2 | 2 | 2 | 2 | 1 | 1 | 2 | 0 | 2 | 2 | 2 | 2 | 2 | 0 | 2 | 2 | 0 | 0 | 2 | 2 | 0 | 0 | 0 | 0 | 1 | 1 | 2 | 2 | 2 | 2 | 2 | 0 |
| Prospective study registered in a trial database | 0 | 0 | 0 | 0 | 0 | 0 | 0 | 0 | 0 | 0 | 0 | 0 | 0 | 0 | 7 | 7 | 0 | 0 | 0 | 0 | 0 | 0 | 0 | 0 | 0 | 0 | 0 | 0 | 0 | 0 | 0 | 0 |
| Validation | 2 | 2 | 2 | 2 | 3 | 3 | 2 | 2 | 2 | 2 | 2 | 2 | 4 | 2 | 5 | 5 | 2 | 2 | 5 | 5 | 2 | 2 | 4 | 2 | 2 | 2 | 3 | 3 | 3 | 3 | 2 | 2 |
| Comparison to ‘gold standard’ | 0 | 0 | 2 | 2 | 0 | 0 | 0 | 0 | 0 | 0 | 0 | 0 | 0 | 0 | 2 | 2 | 2 | 2 | 2 | 2 | 2 | 2 | 0 | 0 | 0 | 0 | 2 | 2 | 2 | 2 | 0 | 0 |
| Potential clinical utility | 2 | 2 | 2 | 2 | 2 | 2 | 2 | 2 | 2 | 2 | 2 | 2 | 2 | 2 | 2 | 2 | 2 | 2 | 2 | 2 | 2 | 2 | 0 | 0 | 0 | 0 | 2 | 2 | 2 | 2 | 2 | 2 |
| Cost-effectiveness analysis | 0 | 0 | 0 | 0 | 0 | 0 | 0 | 0 | 0 | 0 | 0 | 0 | 0 | 0 | 0 | 0 | 0 | 0 | 0 | 0 | 0 | 0 | 0 | 0 | 0 | 0 | 0 | 0 | 0 | 0 | 0 | 0 |
| Open science and data | 0 | 0 | 0 | 0 | 0 | 0 | 0 | 0 | 0 | 0 | 0 | 0 | 0 | 0 | 0 | 2 | 0 | 0 | 0 | 0 | 0 | 0 | 0 | 0 | 0 | 0 | 2 | 0 | 0 | 0 | 0 | 0 |
| total points=36=100% | 16 | 16 | 17 | 17 | 12 | 14 | 13 | 11 | 13 | 13 | 13 | 13 | 15 | 11 | 26 | 28 | 14 | 14 | 19 | 19 | 11 | 11 | 3 | 1 | 10 | 10 | 18 | 16 | 19 | 19 | 12 | 10 |
| Mean score (%) | 44.44 | | 47.22 | | 36.11 | | 33.33 | | 36.11 | | 36.11 | | 36.11 | | 75.00 | | 38.89 | | 52.78 | | 30.56 | | 5.56 | | 27.78 | | 47.22 | | 52.78 | | 30.56 | |

Raters: R1, rater 1, R2, rater 2. Study ID: S1, Limin Zhang, S2, Jeremy Hofmeister, S3, Guanmin Quan, S4, Linna Li, S5, Haoyue Zhang, S6, Tatsat R. Patel, S7, Xing Xiong, S8, Lucas A. Ramos, S9, W. Qiu, S10, Tian-yu Tang, S11, Hui Cui, S12, Manon L. Tolhuisen, S13, Wei Ye, S14, Emily W. Avery, S15, Liang Jiang, S16, Huan Yu.

# Supplemental table 6. Summary of ROB evaluation of studies

| **Study ID** | **Participants** | **Predictors** | **Outcome** | **Analysis** | **Overall** |
| --- | --- | --- | --- | --- | --- |
| Limin Zhang | high | high | low | unclear | high |
| Jeremy Hofmeister | low | low | low | high | high |
| Guanmin Quan | high | high | low | low | high |
| Linna Li | high | high | low | low | high |
| Haoyue Zhang | high | high | low | low | high |
| Tatsat R. Patel | high | high | low | unclear | high |
| Xing Xiong | high | high | low | low | high |
| Lucas A. Ramos | low | low | low | low | low |
| W. Qiu | high | high | unclear | low | high |
| Tian-yu Tang | low | low | low | unclear | unclear |
| Hui Cui | high | high | low | low | high |
| Manon L. Tolhuisen | high | high | high | unclear | high |
| Wei Ye | high | high | low | low | high |
| Emily W. Avery | high | high | low | low | high |
| Liang Jiang | high | high | low | low | high |
| Huan Yu | high | high | low | low | high |

ROB, risk of bias.


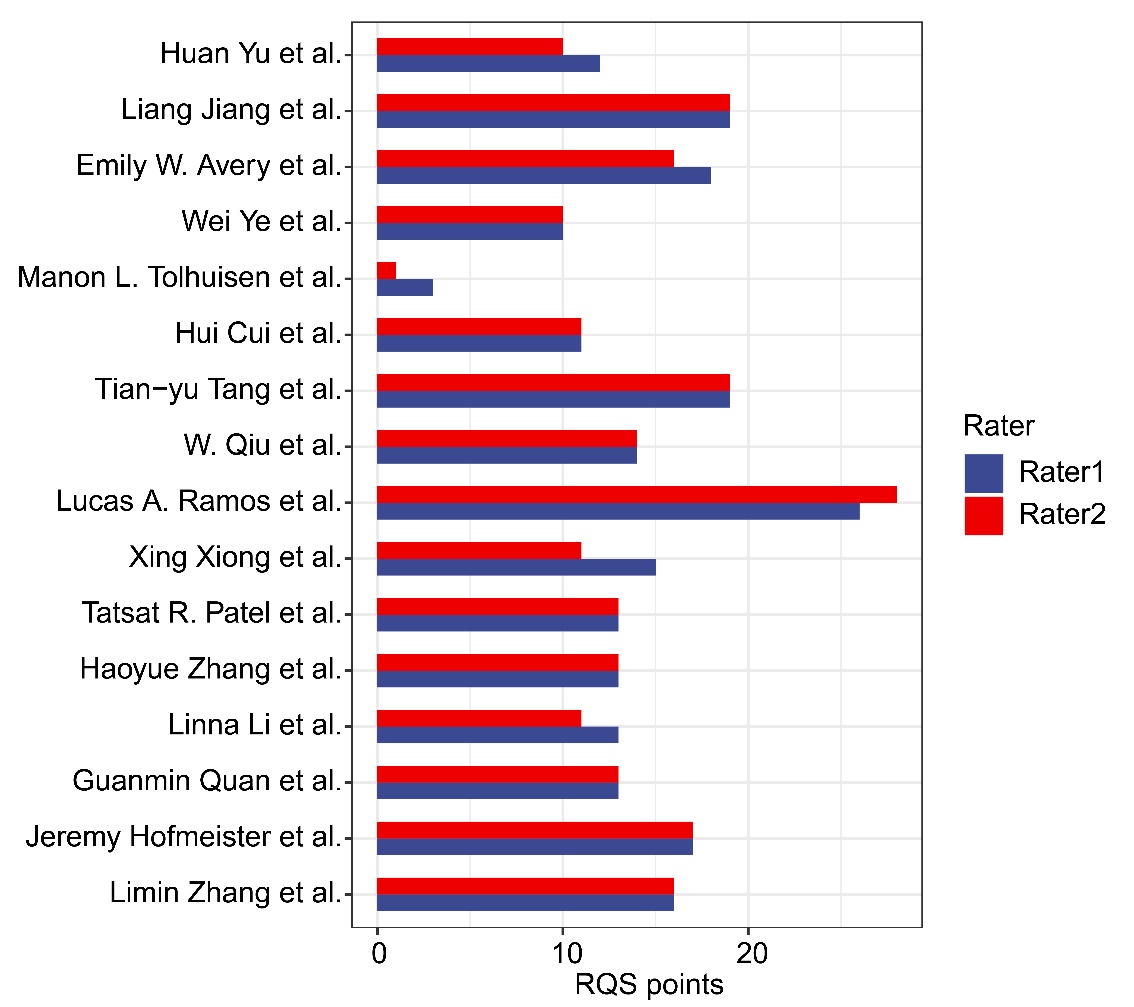


# Supplemental figure 1. The radiomics quality score of included studies by each rater
